# Mediating Effect of Perceived Ageism Experiences Between TikTok Addiction Symptoms and Psychological Distress: A Cross‐Sectional Study

**DOI:** 10.1002/hsr2.73341

**Published:** 2026-10-05

**Authors:** Anthony Rizk, Nelly Kheir, Rabih Hallit, Katia Iskandar, Diana Malaeb, Muna Barakat, Fouad Sakr, Mariam Dabbous, Frederic Harb, Sahar Obeid, Souheil Hallit, Feten Fekih‐Romdhane

**Affiliations:** ^1^ School of Medicine and Medical Sciences Holy Spirit University of Kaslik Jounieh Lebanon; ^2^ Faculty of Medicine Paris‐Saclay University Le Kremlin‐Bicêtre France; ^3^ Department of Infectious Disease Bellevue Medical Center Mansourieh Lebanon; ^4^ Department of Infectious Disease, Notre Dame des Secours University Hospital Center Byblos Lebanon; ^5^ School of Pharmacy Lebanese International University Beirut Lebanon; ^6^ College of Pharmacy Gulf Medical University Ajman United Arab Emirates; ^7^ Department of Clinical Pharmacy and Therapeutics, Faculty of Pharmacy Applied Science Private University Amman Jordan; ^8^ Department of Biomedical Sciences, University of Balamand Faculty of Medicine and Medical Sciences Kalhat Lebanon; ^9^ School of Arts and Sciences, Social and Education Sciences Department Lebanese American University Jbeil Lebanon; ^10^ Department of Psychology, College of Humanities Effat University Jeddah Saudi Arabia; ^11^ The Tunisian Center of Early Intervention in Psychosis, Department of Psychiatry “Ibn Omrane” Razi Hospital Manouba Tunisia; ^12^ Faculty of Medicine of Tunis Tunis El Manar University Tunis Tunisia

**Keywords:** anxiety, cross‐sectional study, depression, perceived ageism experiences, TikTok addiction

## Abstract

**Background and Aims:**

TikTok addiction has been linked to depression and anxiety, yet mechanisms underlying this relationship remain underexplored. Based on the Social Comparison and Uses and Gratifications theories, this study examined whether perceived ageism experiences mediate the relationship between TikTok addiction and depression and anxiety among Lebanese adults. The research also explores, as a theoretical possibility, whether TikTok's algorithm may be associated with greater perceived ageism experiences, which in turn would relate to psychological distress.

**Methods:**

A cross‐sectional survey was conducted among 855 participants (mean age 24.39; 76.7% females) using the TikTok Addiction Test, WHO Ageism Scale, and PHQ‐4. Data were analyzed using SPSS and mediation analysis via PROCESS Macro controlling for demographic and health covariates, such as gender, education level and a history of mental health diagnosis. The mediation analysis included 735 participants with complete data for all variables entered into the model.

**Results:**

TikTok addiction was positively associated with psychological distress. Perceived ageism experiences partially mediated this relationship (unstandardized indirect effect: B = 0.0149, BootSE = 0.005, 95% percentile bootstrapped CI [0.0058, 0.0255]). Higher TikTok addiction symptoms were associated with greater perceived ageism experiences, which were in turn associated with higher psychological distress levels. Demographic analysis showed that females, those with lower levels of education and individuals with a history of mental health diagnosis reported higher psychological distress. Age was negatively correlated with psychological distress, showcasing higher vulnerability among younger individuals.

**Conclusion:**

Findings highlight perceived ageism experiences as a small but statistically significant mediator between addictive digital media use and psychological distress. The study emphasizes the need for culturally sensitive mental health and digital literacy interventions, as they aim to support the deconstruction of idealized youthful standards, particularly with the Lebanese context where digital media serves as escapism.

**Abbreviations**
BMI: body mass index
CI: confidence interval
HCI: Household Crowding Index
MENA: Middle East and North Africa
PHQ‐4: Patient Health Questionnaire‐4
SD: standard deviation
SE: standard error
SPSS: statistical package for the social sciences
STROBE: strengthening the reporting of observational studies in epidemiology
TAT: TikTok addiction test
WHO: World Health Organization

## 1 Introduction

The proliferation of social media has altered communication, self‐expression, and lifestyle across the globe, particularly among young adults [1, 2]. Platforms such as TikTok, Instagram, and Facebook provide users with highly engaging content, facilitating both social interaction and identity formation. While social media offers benefits, including social connectedness and access to information, mounting evidence links excessive use—often conceptualized as social media addiction—to negative psychological outcomes [3, 4, 5]. Meta‐analyses and systematic reviews demonstrate that high engagement with social media correlates with elevated symptoms of depression, anxiety and loneliness [1, 6]. In the MENA region, research indicates that social media addiction is prevalent and significantly predicts depression and anxiety [7, 8].

Among social media platforms, TikTok has emerged as a unique case due to its algorithmic design, personalized short‐form videos, and immersive content delivery system [9, 10]. These features enhance user engagement but also increase the risk of addictive use, characterized by compulsive scrolling, difficulty disengaging, and preoccupation with content [11, 12]. Systematic reviews indicate that problematic TikTok use is consistently linked to elevated depression, anxiety, and reduced overall well‐being [9, 10, 11, 12]. Unlike traditional social media, TikTok's algorithm amplifies exposure to highly stimulating content, potentially increasing upward social comparison and reinforcing maladaptive engagement patterns [13].

The link between TikTok use and psychological outcomes can be explained through established theoretical frameworks. Social Comparison Theory posits that individuals evaluate themselves against others' behaviors or attributes, often leading to feelings of inadequacy or lower self‐esteem [2, 14]. On platforms like TikTok, exposure to idealized content, influencer lifestyles, and peer‐generated media can exacerbate anxiety and depressive symptoms through upward comparisons [3, 4]. Uses and Gratifications Theory further clarifies why TikTok engagement can become excessive. This theory suggests that individuals actively seek media to satisfy psychological needs, including entertainment, social interaction, and self‐expression [15, 16]. When gratifications are immediate and highly reinforcing, as with TikTok's algorithmic feed, individuals may develop addictive patterns, intensifying exposure to negative psychosocial effects [3, 15]. Together, these frameworks provide a foundation for understanding both the direct effects of social media on mental health and the potential mechanisms through which these effects are amplified.

Beyond general psychosocial distress, a robust theoretical rationale connects problematic TikTok engagement specifically to the reporting of perceived ageism experiences. Grounded in Digital Ageism and Content‐Specific Exposure Frameworks, TikTok's algorithmic architecture is systematically biased toward vitality and youth‐centric validation and esthetic perfectionism [17] (Erdenebat & Veloso da Silva, 2026). The platform's rapid, short‐form visual delivery relies heavily on promoting youthful esthetics while routinely rendering older adults invisible or associated solely with decline [18]. Consequently, individuals exhibiting symptoms of problematic and/or compulsive use experience intensified, passive doses of age‐biased media content. Through repeated digital exposure to these pervasive standards, users may become increasingly likely to notice and report ageism in their online environment, a distinct construct from holding ageist attitudes oneself [19].

Another potential correlational pathway underlying the relationship between excessive TikTok use and mental health outcomes on which this study focuses is ageism. Ageism, defined as prejudice, stereotyping, or discrimination based on age, is increasingly recognized in digital media contexts [19, 20, 21]. Social media can both perpetuate and challenge ageist attitudes, as online content often marginalizes older adults while idealizing youth and beauty [17, 18]. Distinct from holding ageist attitudes oneself, perceiving or experiencing ageism has also been linked to negative mental health outcomes, including increased anxiety and depressive symptoms [22, 23]. Experimental research indicates that social media‐based interventions can successfully reduce negative stereotypes and promote more positive perceptions of aging [24].

Recent research suggests that ageism may serve as a correlational factor in the relationship between social media use and mental health outcomes. Problematic engagement with TikTok can increase exposure to ageist content, which may be perceived and reported as ageism and contribute to elevated depression and anxiety [12, 24, 25]. Although mediation studies are limited, preliminary evidence supports the notion that experiences of ageism can partially explain why excessive social media use adversely affects psychological well‐being [19, 21]. By examining perceived ageism experiences as a potential mediator, research can move beyond simple correlational associations and explore the potential explanatory pathways linking TikTok addiction to mental health outcomes.

Although traditional theoretical arguments about ageism are framed around older populations, youth‐idealizing digital content might be significantly lowering the age at which individuals feel assessed against their beauty and vitality standards, normalizing earlier anti‐aging concerns among young adults. Findings among a young sample might help capture anticipatory exposure to age‐based social evaluation rather than lived age discrimination, supporting its relevance as a mediator.

The MENA region presents a distinct sociocultural and digital landscape in which social media use is pervasive [7]. Platforms like TikTok are widely adopted among youth and adults alike, often serving as primary sources of social interaction and information. In Lebanon, high internet penetration and smartphone use have created an environment in which social media engagement is integral to daily life, yet mental health concerns—including depression and anxiety—remain prevalent and under‐addressed [8, 26]. Lebanese cultural factors, such as strong intergenerational family structures and respect for elders, intersect with modern social media consumption, potentially influencing attitudes toward aging [26, 27]. Moreover, social media platforms may amplify societal pressures, increase upward comparisons, and expose users to content that subtly reinforces ageist stereotypes. Regional studies in Lebanon and other MENA countries highlight associations between problematic social media use and poor psychological well‐being, reinforcing the importance of context‐specific research [7, 8]. Therefore, the main objective of our study was to explore the direct and indirect effect of TikTok addiction symptoms of depression‐anxiety through perceived ageism experiences. Understanding how these dynamics operate within Lebanon is essential for informing public health interventions, digital literacy programs, and strategies to reduce experiences of perceived ageism, contributing to healthier online engagement and improved mental health outcomes.

## 2 Methods

### 2.1 Study Design and Setting

This study employed a cross‐sectional design, using an anonymous online questionnaire that was conducted between July and August 2025. Lebanese adults aged 18 and above, utilize TikTok, and have provided voluntary acceptance to participate in this study were the targeted population to who the survey was sent to. The survey was hosted on Google Forms in the Arabic language, it disseminated to the target population via various social media platforms following revisions done by the Arabic‐speaking researchers to ensure clarity and relevance. Participants were first given an overview of the study's purpose and participation anonymity. This section also provided an estimation of the time required to complete the survey (15–20 min) and emphasized participants' right to withdraw from the study at any time. Following this part, informed consent was obtained through a mandatory preliminary question, participants that consented were redirected to the main survey. To ensure data integrity, the survey restricted each participant to a single submission. This study's ethical approval was obtained via the Institutional Review Board (IRB) of the School of Pharmacy, Lebanese International University (approval reference: 2025ERC‐025‐LIUSOP), prior to data collection, which took place in 2025 as noted above.

This study is reported in accordance with the Strengthening the Reporting of Observational Studies in Epidemiology (STROBE) statement for cross‐sectional studies [28] and follows the statistical reporting recommendations of Assel et al. (2018) for clinical research.

### 2.2 Minimum Sample Size

Minimum sample size calculation was done with the Fritz and MacKinnon's [29] power analysis recommendations for mediation analysis. With expectations of small effect sizes for the indirect pathways, a statistical power of 0.80 and an *α* = 0.05, the minimum required sample size was around 462 participants [29].

### 2.3 Measures

The questionnaire started by collecting demographic data of participants, including age, gender, marital status, home environment, and educational level, as well as some health habits like smoking, alcohol, and cannabis use. Furthermore, participants were asked to indicate by yes or no whether they have a mental health diagnosis.

#### 2.3.1 TikTok Addiction Test (TAT)

A brief self‐report measure developed to assess symptoms of problematic TikTok use. Consisting of 6 items, rated on a 5‐point Likert scale ranging from 0 (very rarely) to 4 (very often), measuring experiences over the past 12 months. Higher scores indicate greater addiction risk [8] (Cronbach's α in this study = 0.88).

#### 2.3.2 The WHO Ageism Scale

A 5‐item scale used for assessing perceived ageism from an ‘experiences' perspective. Scoring is done via a 5‐point Likert scale with scoring from 1 (Strongly agree) to 5 (Strongly disagree) and for other negatively worded items, scoring goes from 5 (Strongly agree) to 1 (Strongly disagree), with “Don't know or not applicable” answer. Higher scores indicate greater perceived ageism experiences. The Arabic version was used for this study [30] (Cronbach's α in this study = 0.79).

#### 2.3.3 The Patient Health Questionnaire (PHQ‐4)

An ultra‐brief 4‐item scale used to assess psychological distress symptoms over the last 2 weeks through 2 items each [31]. Answers range from 0 (not at all) to 3 (almost every day), with higher scores indicating greater psychopathology. The Arabic validated version was used in this study [32] (Cronbach's α in this study = 0.87).

### 2.4 Statistical Analysis

Data analysis was performed using SPSS software version 27. The normality of the psychological distress scores were considered normally distributed since the skewness and kurtosis values varied between ‐1 and +1. Student t or Welch tests were used to compare two means, ANOVA test to compare three or more means, and Pearson's test to correlate two continuous variables. The mediation analysis was performed using PROCESS Macro version 4.2 Model 4. Four pathways were computed: Pathway A from TikTok addiction and perceived ageism experiences, Pathway B from ageism and psychological distress, and Pathways C and C' reflecting the total and direct association of TikTok addiction on depression and anxiety respectively. Assumptions underlying the regression models were evaluated before interpreting the mediation analysis. Linearity and homoscedasticity were assessed by inspecting plots of standardized residuals against standardized predicted values, while residual normality was evaluated using a histogram and normal P‐P plot. Multicollinearity was assessed using tolerance and variance inflation factor (VIF) values. The indirect effect was estimated using 5,000 bootstrap samples and a 95% percentile bootstrap confidence interval. The mediation analysis included 735 participants with complete data for the exposure, mediator, outcome, and covariates. Covariates entered in the model were those that showed a *p* < 0.25 in the bivariate analysis. Significance was considered for a *p* < 0.05.

The mediation analysis (Pathways A, B, C, and C') constituted the prespecified primary analysis testing the study's central hypothesis. Percentile bootstrap 95% confidence intervals for the indirect effect were derived from 5,000 bootstrap samples. Analyses were performed using IBM SPSS Statistics, version 27.0 (IBM Corp., Armonk, NY, USA) and the PROCESS macro for SPSS, version 4.2 (Model 4 [33]). Statistical terms and abbreviations used throughout this report are defined as follows: B, unstandardized regression coefficient; β, standardized regression coefficient; SE, standard error; CI, confidence interval; SD, standard deviation; BootSE, bootstrap standard error; and Boot CI, percentile bootstrapped confidence interval. Potential covariates available in the dataset were initially examined in bivariate analyses. Variables associated with psychological distress at *p* < 0.25 were subsequently included in the mediation model. This deliberately liberal threshold was used to reduce the likelihood of prematurely excluding potentially relevant covariates based on the conventional *p* < 0.05 criterion. Accordingly, the final model was adjusted for gender, marital status, education, mental health diagnosis, age and BMI. This screening procedure represented a pragmatic model‐building strategy and was not intended to identify an exhaustive or causally sufficient set of confounders. All statistical tests were two‐sided, and the a priori level of statistical significance was set at *α* = 0.05. This report follows the Statistical Analyses and Methods in the Published Literature (SAMPL) guidelines [34] and the recommendations of Assel et al. (2018) for the reporting of statistics in clinical research.

## 3 Results

In total, 855 participants completed the questionnaire, with 656 (76.7%) females and a mean age of 24.30 years. The full description of the sample is in Table 1.

**Table 1:** Sociodemographic and other characteristics of participants (*N* = 855).

	*N* (%)
Gender	
Male	199 (23.3%)
Female	656 (76.7%)
Marital status	
Single, divorced, widowed	689 (80.6%)
Married, in a relationship	166 (19.4%)
Living status	
Alone	22 (2.6%)
With family or partner	822 (96.1%)
With others	11 (1.3%)
Education level	
School level	160 (18.7%)
University level	695 (81.3%)
Cigarettes	
No	727 (85.0%)
Yes	128 (15.0%)
Alcohol	
No	787 (92.0%)
On occasions	61 (7.1%)
Every week	7 (0.8%)
Used cannabis in your life	
No	843 (98.6%)
Yes	12 (1.4%)
Diagnosed with mental health	
No	826 (96.6%)
Yes	29 (3.4%)
	**Mean ± SD**
Age (years)	24.39 ± 8.86
Household Crowding Index (HCI)	1.21 ± 0.62
Body Mass Index (BMI)	23.89 ± 4.57
Perceived Ageism experiences	7.73 ± 4.45
TikTok addiction	4.99 ± 5.00
Psychological distress	3.65 ± 3.14

### 3.1 Bivariate Analysis

Being female, having school‐level vs university‐level education and having a mental health diagnosis were significantly associated with higher psychological distress scores (Table 2). Furthermore, greater TikTok addiction and higher levels of perceived ageism experiences were significantly associated with higher psychological distress scores, whereas older age was significantly associated with lower psychological distress scores (Table 3).

**Table 2:** Bivariate analysis of factors associated with psychological distress scores.

	Mean ± SD	*t*	df	*p*	Effect size (Cohen's d or Eta‐squared)
Gender		3.52	853	**< 0.001**	0.285
Male	2.97 ± 2.97				
Female	3.86 ± 3.16				
Marital status		1.33	853	0.19	0.115
Single, divorced, widowed	3.72 ± 3.15				
Married, in a relationship	3.36 ± 3.11				
Living status		0.40	2, 852	0.67	0.001
Alone	3.18 ± 2.50				
With family or partner	3.66 ± 3.15				
With others	4.18 ± 3.63				
Education level		2.97	853	**0.003**	0.260
School level	4.31 ± 3.25				
University level	3.50 ± 3.10				
Cigarettes		0.29	853	0.77	0.028
No	3.64 ± 3.08				
Yes	3.73 ± 3.45				
Alcohol		0.25	2, 852	0.78	0.001
No	3.64 ± 3.09				
On occasions	3.90 ± 3.79				
Every week	3.29 ± 2.81				
Used cannabis in your life		0.64	11.14	0.53	0.274
No	3.64 ± 3.12				
Yes	4.50 ± 4.62				
Diagnosed with mental health		5.13	29.23	**< 0.001**	1.211
No	3.53 ± 3.04				
Yes	7.24 ± 3.86				

**Table 3:** Pearson correlation matrix.

	1	2	3	4	5	6
1. Psychological distress	1					
2. TikTok addiction	0.35***	1				
3. Perceived ageism experiences	0.23***	0.17***	1			
4. Age	−0.08*	−0.17***	−0.03	1		
5. Household Crowding Index	0.04	0.03	0.04	−0.001	1	
6. Body Mass Index	0.05	−0.07	0.004	0.35***	−0.01	1

**p < 0.01

* *p* < 0.05

*** *p* < 0.001.

Bold numbers indicate significant *p* value. *p* values are reported per HSR convention: < 0.001 for *p* < 0.001, to the nearest thousandth for 0.001 ≤ *p* < 0.01, and to the nearest hundredth for *p* ≥ 0.01 (strikethrough values show original reporting; bold values show corrected formatting).

### 3.2 Analysis of Mediation

Assumptions of linearity, homoscedasticity and residual normality were all met. No evidence of problematic multicollinearity was identified: tolerance values ranged from 0.387 to 0.974 and VIF values between 1.027 and 2.586. Visual inspection of the residual histogram and normal P‐P plot indicated that the residuals were approximately normally distributed.

The mediation analysis taking the psychological distress scores as the dependent variable, was adjusted over the following covariates: gender, marital status, education level, diagnosed with mental health, age and body mass index. The analysis included 735 participants with complete data for all variables entered into the model. A statistically significant but small indirect association was found between TikTok addiction and psychological distress through perceived ageism experiences (unstandardized indirect effect: B = 0.0149, Boot SE = 0.005; 95% percentile bootstrap CI [0.0058, 0.0255]; completely standardized indirect effect: *β* = 0.0243, Boot SE = 0.0083; 95% percentile bootstrap CI [0.0094, 0.0422]). Higher TikTok addiction was significantly associated with greater perceived ageism experiences, which in turn was significantly associated with higher psychological distress. Finally, the direct association between TikTok addiction and psychological distress remained significant (B = 0.19, SE = 0.02, *β* = 0.31, *p* < 0.001; Figure 1).

**Figure 1:**
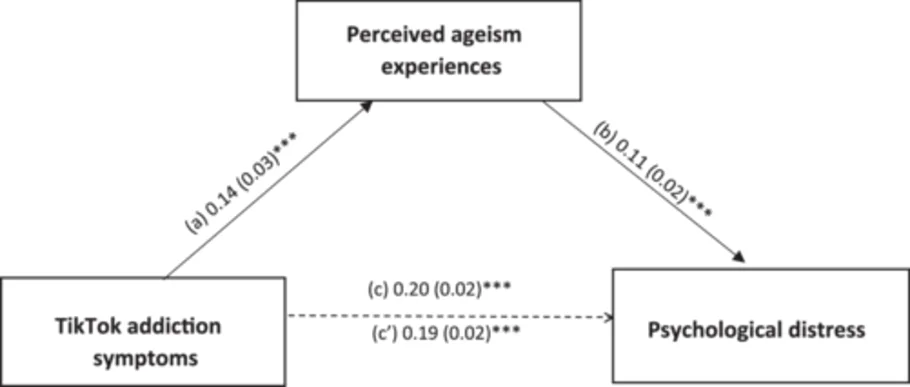
Mediation model taking perceived ageism experiences as a mediator between TikTok addiction symptoms and psychological distress. (a) Relation between TikTok addiction and perceived ageism experiences (*R* ^2^ = 0.032); (b) Relation between perceived ageism experiences and psychological distress (*R* ^2^ = 0.206); (c) Total effect of TikTok addiction on psychological distress (*R* ^2^ = 0.183); (c') Direct effect of TikTok addiction on psychological distress. The numbers represent regression coefficients and their standard errors. ***p < 0.001, *N* = 735. The mediation was based on participants with complete data for all variables included in the model. Coefficients are unstandardized regression weights (B) with standard errors in parentheses; the model was adjusted for gender, marital status, education level, mental health diagnosis, age, and body mass index (BMI). CI, confidence interval; *R* ^2^, coefficient of determination. The 95% percentile bootstrap confidence interval for the indirect effect of TikTok addiction on psychological distress via perceived ageism was [0.0058, 0.0255], based on 5000 bootstrap samples.

## 4 Discussion

The current paper studied the association of perceived ageism experiences on the relationship between TikTok addiction symptoms and anxiety‐depression among Lebanese adults. Results showed that TikTok addiction symptoms are significantly associated with higher levels of psychological distress. These findings align with the international body of research on the negative correlation between problematic social media use and poorer mental health outcomes [35, 36, 37]. Additionally, perceived ageism experiences were significantly correlated with both variables. This may be particularly relevant given the young mean age of the sample (24.39 years), as perceived ageism experiences in this group plausibly reflect early exposure to youth‐based social comparison online rather than the effects of chronological aging itself.

The direct relationship between TikTok addiction and psychological distress supports behavioral addiction research. Addictive social media use has been linked to increased rumination and emotional instability [38, 39]. Social media overuse is also associated with decreased real‐world social interaction quality and reduced life satisfaction [40]. Short‐form video platforms such as TikTok may show similar or stronger patterns compared to traditional social media, given their rapid content turnover and high sensory stimulation [11]. Uses and Gratifications Theory helps explain how social media users actively seek these platforms to satisfy their psychological needs (e.g., entertainment, social validation and emotional regulation) [16, 41]. Social media addiction and other addictions have many similarities, including mood modification, tolerance and symptoms of withdrawal [42, 43]. Systematic reviews have constantly shown that problematic social media use is associated with increased loneliness and emotional dysregulation [36, 44].

The association of perceived ageism experiences can be understood through Social Comparison Theory [14], which suggests that individuals evaluate their self‐worth by comparing themselves to socially valued standards of age and beauty. Photographic social media platforms are linked to higher upward social comparisons via the constant exposure of ideal representations of appearance, lifestyle and youthfulness [45, 46]. Research consistently shows that upward social media comparisons correlate with symptoms of depression, anxiety and low self‐esteem [47, 48]. Reporting greater perceived ageism experiences may reflect increased exposure to a cultural context that equates youth with social value, attractiveness, and productivity, rather than the internalization of these standards as personal beliefs [19]. The Stereotype Embodiment Theory proposes that individuals internalize cultural stereotypes about aging throughout their lifespan, which is associated with psychological and physical health outcomes [49]. It should be noted that while this theory concerns the internalization of stereotypes as self‐relevant beliefs, the present study measured perceived exposure to and experience of ageism, rather than the extent to which participants had internalized these stereotypes themselves. Longitudinal research has shown that low perceived ageism experiences correlates with better mental health and longevity, whereas higher perceived ageism relates to increased depressive symptoms and cognitive decline risk [50]; B [19]. Exposure to digital platforms focused on youth is associated with ageism [51], which has been consistently linked with worsened depressive symptoms, lower self‐esteem and social withdrawal [52, 53]. The observed correlation between perceived ageism experiences and psychological distress in the present sample is consistent with the broader literature linking anti‐aging beauty standards on social media to ageism and self‐objectification; however, self‐objectification was not directly measured here, and this interpretation should be regarded as consistent with, rather than confirmed by, the current data [54, 55].

The findings may be usefully considered, but were not directly assessed in this study within the Lebanese context, where collectivist social structures and economic instability shape how appearance and digital media use are experienced. In the collectivist societies on the MENA region, social identity and family reputation are tightly linked to appearance and social presentation [56, 57]. Given Lebanon's ongoing socioeconomic instability, increased reliance on social media as a coping mechanism is also plausible [38, 58], although this pathway, like the appearance‐related pressures described above, was not directly measured here.

### 4.1 Limitations

This study has several limitations that must be acknowledged. First, the cross‐sectional design and convenience sample recruited through social media platforms prevent establishing causal relationships between TikTok addiction, perceived ageism experiences, and psychological distress. Furthermore, the sample composition comprised of 76.7% women, had a mean age of 24.3 years, and included more than 81% of participants who held a university degree, substantially limiting the generalizability of the findings to the broader Lebanese adult population. Second, reliance on self‐reported measures may introduce response bias and social desirability effects. Third, covariate selection was partly data‐driven, as measured variables associated with psychological distress at *p* < 0.250 in the bivariate analyses were entered in the mediation model. Although this liberal threshold was intended to avoid prematurely excluding potentially relevant variables, bivariate associations do not necessarily identify confounders, and the resulting adjustment set may not fully represent the causal structure linking the study variables. Unmeasured confounding variables such as socioeconomic status and personality traits were not included. In addition, other theoretically relevant factors, including general health status, social comparison, self‐esteem, body dissatisfaction, fear of missing out, emotion regulation, and perceived loneliness were not measured. Consequently, residual confounding cannot be excluded, and the estimated indirect association should not be interpreted as establishing a causal mediating mechanism. Additionally, the study did not distinguish between different types of TikTok content exposure, which might have different psychological effects. Finally, the additional interpretive mechanisms proposed in the Discussion (including algorithmic reinforcement, aging‐related anxiety, appearance‐related pressure, self‐objectification, and Lebanon‐specific sociocultural dynamics) were not directly measured in this study; they were drawn from the broader literature to contextualize, rather than confirm, the observed associations. Future longitudinal studies incorporating validated measures of these constructs are needed to test them directly.

### 4.2 Implications and Future Directions

These findings highlight perceived ageism as a relevant, though modest, correlate in digital mental health research, suggesting that the psychological toll of social media is associated not only with duration of use but also with greater perceived ageism experiences. Public health interventions should focus on social media literacy, promotion of realistic aging expectations, and psychological resilience against pressures, particularly by the education of users on the artificiality of social media's beauty standards that exacerbate “ageism.” Whether cognitive‐behavioral interventions targeting perceived ageism experiences and maladaptive comparison processes could help reduce the psychological impact of addictive TikTok use remains a promising question for future intervention research, particularly given that the standardized indirect effect observed here was small (β = 0.0243) and maladaptive comparison was not directly measured in this study.

From a research perspective, these outcomes highlight the need for longitudinal studies to examine temporal direction and potential causal relationships between addictive scrolling and the development of perceived ageism experiences, particularly within the Lebanese context where digital platforms act as an emotional coping mechanism via escapism. Additionally, future directions should involve algorithmic management and control to ensure that social media platforms (e.g., TikTok) foster a more inclusive and mentally healthy digital ecosystem, especially among older demographics.

## 5 Conclusion

In summary, this research shows that TikTok addiction symptoms are strongly associated with psychological distress among Lebanese adults, with perceived ageism accounting for a small but statistically significant indirect association between the two. These results are consistent with a growing body of global evidence that links problematic social media habits to reduced mental well‐being. Within the unique sociocultural framework of Lebanon, specific pressures regarding public image and aging may plausibly contribute to psychological vulnerability, although this pathway was not directly assessed in the present study. From a clinical and public health perspective, these findings suggest that interventions should focus on strengthening digital media literacy and cognitive resilience, helping users manage the perceived ageism experiences and social pressures they encounter online. While this cross‐sectional study offers a vital snapshot of the current situation, future longitudinal research will be essential to fully map out how these behaviors evolve over time.

## Author Contributions

**Anthony Rizk:** writing – review and editing, writing – original draft. **Nelly Kheir:** formal analysis, writing – review and editing, methodology. **Rabih Hallit:** visualization, validation, writing – review and editing. **Katia Iskandar:** validation, visualization, writing – review and editing. **Diana Malaeb:** validation, visualization, writing – review and editing. **Muna Barakat:** validation, visualization, writing – review and editing. **Fouad Sakr:** data curation, writing – review and editing, visualization. **Mariam Dabbous:** data curation, writing – review and editing, visualization. **Frederic Harb:** visualization, writing – review and editing, validation. **Sahar Obeid:** writing – review and editing, visualization, validation. **Souheil Hallit:** formal analysis, supervision, project administration, writing – review and editing, writing – original draft, methodology, software. **Feten Fekih‐Romdhane:** conceptualization, visualization, validation, writing – review and editing, investigation.

## Funding

The authors have nothing to report.

## Ethics Statement

The ethics approval for the study was granted by the School of Pharmacy at the Lebanese International University (2025ERC‐025‐LIUSOP). When filling out the online form, each participant provided written informed consent. All methods were performed in accordance with the relevant guidelines and regulations (in accordance with the Declaration of Helsinki).

## Conflicts of Interest

The authors declare no conflicts of interest.

## Transparency Statement

The Corresponding author (F.F.R.) affirms that this manuscript is an honest, accurate, and transparent account of the study being reported; that no important aspects of the study have been omitted; and that any discrepancies from the study as planned (and, if relevant, registered) have been explained.

## Data Availability

Because of ethical committee constraints, none of the data collected or analyzed during this study are publicly available. However, the corresponding author (S.H.) may make the data available upon reasonable request.
